# P04.56. Acupoint versus non-acupoint injection of antiviral treatment for chronic hepatitis B: a systematic review of randomized controlled trials

**DOI:** 10.1186/1472-6882-12-S1-P326

**Published:** 2012-06-12

**Authors:** J Liu, L Ma, M Han, C Liu, X Pan

**Affiliations:** 1 Beijing University of Chinese Medicine, Beijing, China; 2 Tianjin University of Traditional Chinese Medicine, Tianjin, China

## Purpose

Antiviral drugs are standard treatment for chronic hepatitis B. In traditional Chinese medicine practice, administration of drugs through acupuncture point (acupoint) is considered better than intramuscular injection or intravenous infusion. In this review, we evaluated comparative effects and safety of antiviral drugs through acupoint injection versus other administration for treatment of chronic hepatitis B.

## Methods

We systematically searched PubMed, the Cochrane Library, the Cochrane Hepato-Biliary Group Trial Register, and four Chinese electronic databases through March 2011 to identify randomized controlled trials (RCTs) comparing acupoint injection with intramuscular, subcutaneous injection or intravenous infusion for at least three months for treatment of chronic hepatitis B. The quality of the RCTs was assessed using the Cochrane risk of bias tool and data were synthesized using RevMan 5.

## Results

Ten RCTs involving 1618 people with chronic hepatitis B were included. Three of 10 RCTs were rated as having low risk of bias and the remaining trials as high or uncertain risk of bias. Acupoint injection of interferon-alpha showed significantly better effect on the loss of serum HBeAg than intramuscular injection (RR 1.37, 95% confidence interval (CI) 1.10 to 1.70; 3 trials). Similarly, acupoint injection of interferon-alpha was better than intravenous infusion for loss of serum HBeAg (RR 2.38, 95% CI 1.17 to 4.83; 3 trials). However, there was no significant difference between acupoint and intramuscular injection of interferon-alpha in terms of the loss of serum HBsAg or HBV DNA. Acupoint injection of poly I-C, or matrine showed beneficial effects on loss of serum HBeAg compared with intramuscular injection. Acupoint injection of antiviral drugs did not report extra adverse effects from the included trials.

## Conclusion

Acupoint injection of antiviral drugs for three months treatment for chronic hepatitis B may have more beneficial effects than intramuscular or intravenous injection. However, the findings need to be confirmed in large, rigorous randomized trials.

